# Collecting Psycholinguistic Response Time Data Using Amazon Mechanical Turk

**DOI:** 10.1371/journal.pone.0116946

**Published:** 2015-03-30

**Authors:** Kelly Enochson, Jennifer Culbertson

**Affiliations:** 1 Linguistics Program, Department of English, George Mason University, Fairfax, Virginia, United States of America; 2 School of Philosophy, Psychology and Language Sciences, University of Edinburgh, Edinburgh, United Kingdom; Mälardalen University, SWEDEN

## Abstract

Researchers in linguistics and related fields have recently begun exploiting online crowd-sourcing tools, like Amazon Mechanical Turk (AMT), to gather behavioral data. While this method has been successfully validated for various offline measures—grammaticality judgment or other forced-choice tasks—its use for mainstream psycholinguistic research remains limited. This is because *psycholinguistic* effects are often dependent on relatively small differences in response times, and there remains some doubt as to whether precise timing measurements can be gathered over the web. Here we show that three classic psycholinguistic effects can in fact be replicated using AMT in combination with open-source software for gathering response times client-side. Specifically, we find reliable effects of subject definiteness, filler-gap dependency processing, and agreement attraction in self-paced reading tasks using approximately the same numbers of participants and/or trials as similar laboratory studies. Our results suggest that psycholinguists can and should be taking advantage of AMT and similar online crowd-sourcing marketplaces as a fast, low-resource alternative to traditional laboratory research.

## Introduction

In recent years, researchers in linguistics and related fields have begun using Amazon Mechanical Turk (AMT; www.mturk.com) to conduct behavioral experiments including acceptability and similarity judgment tasks [1–3], sentence completion tasks [3–5], and artificial language learning tasks [5–7]. AMT is an online crowd-sourcing marketplace that allows researchers to post experiments, easily recruit large numbers of participants from broad demographic groups, and provide compensation in an automated way. In contrast to traditional methods of data collection, which require a sizeable pool of undergraduate participants run consecutively over the course of several weeks or even months, tasks like those listed above can typically be completed on AMT within a few days at a much lower cost. Compared to the relatively homogeneous makeup of undergraduate students, the more diverse population available on AMT may also increase the generalizability of any findings [8].

AMT’s pre-designed templates can be easily used to collect measures of end-state processing or knowledge like acceptability judgments or forced-choice responses for simple experimental designs. However, research in *psycholinguistics* often uses response time measures to capture the effects of processing incrementally as they happen. To date, AMT has not been widely used to collect such data. This is in part due to concerns about collecting responses with millisecond accuracy; differences in response times across devices or servers may introduce a source of variability that could obscure the kinds of small yet meaningful results that can characterize important psycholinguistic effects. As we discuss in more detail below, there is reason to believe that web-based methods can in fact produce reliable effects when this level of precision timing is required. For example, a number of classic effects from cognitive psychology have recently been replicated using AMT [9]. Here we seek to extend the record of successful replication to three important effects in psycholinguistics: (i) faster processing of pronouns compared to DPs, (ii) processing costs associated with filler gaps in *wh*-fronted constructions, and (iii) agreement attraction.

Success in using web-based methods for collecting psycholinguistic response time data has been reported in several recent studies (e.g., [5,10–12]). Keller et al. [10] replicated laboratory self-paced reading results concerning parsing ambiguity using WebExp, a system for conducting psycholinguistic experiments over the web. Also using WebExp, Demberg [12] replicated the well-known psycholinguistic effect of subject-object relative clause asymmetry using data collected over the web. Ibex Farm provides a similar platform for hosting and running psycholinguistic experiments [13], and researchers have recently begun using these platforms more regularly for self-paced reading tasks. Studies such as these have demonstrated the effectiveness of gathering response time data over the web, and the current study hopes to extend this line of research by systematically replicating a number of laboratory experiments using comparable stimuli, trial numbers, and sample sizes. While our main aim is to show that AMT, and the web in general, can be fruitfully used to conduct research in this field, we also note the value of replication studies more generally [14,15].

### Collecting response time data over the web using AMT

AMT allows researchers and other requesters to post *human intelligence tasks*, or HITs, to be completed by workers over the web for a fee. Common types of HITs include transcribing audio, identifying images in photos, or duplicating data. Depending on the length of time the task requires, workers are typically paid somewhere from $.05 for a quick 30-second task to $1.00 or more for longer tasks. HITs are most straightforwardly derived from the templates provided by AMT, which include surveys, translation tasks, rating (Likert scale) tasks, and picture tagging tasks. These templates are comprised of a single static HTML page, and thus are ideal for single trial tasks or those in which the participant can scroll down a page to view subsequent items. However, for tasks which require multiple trials presented one at a time to participants, dynamic updates can be achieved using JavaScript or Flash.

The experiments we will be reporting here involve not just dynamic trial presentation, but the collection of millisecond response time data. The most reliable way to collect response times from participants over the web is to record them *locally* on the participant’s computer (often called client-side) and then collate them on AMT once the task is completed. Previous research using JavaScript suggests that this method is precise enough to capture a number of classic effects in cognitive psychology (e.g., the Stroop effect, the Flanker effect, Task-switching effects, etc.), even when the tasks require sustained attention and complex instructions [9]. A recently developed Flash-based program called ScriptingRT [16] provides free, open-source software (http://reactiontimes.wordpress.com) and templates for response time experiments. The software includes functionality allowing for millisecond precision in the visual display of words or images for priming tasks and the recording of response times for lexical decision or self-paced reading tasks. This software has been validated using the Stroop Test; laboratory results were replicated with only slightly higher variability in response times. Here we use ScriptingRT to replicate three important effects reported in the psycholinguistics literature, which we describe in detail below.

### Three classic effects in psycholinguistics

**PRONOUN VS. DP PROCESSING**. A number of psycholinguistic studies have shown that referring expressions that differ in definiteness are processed or judged differently. This has been claimed to be the result of relative ease of accessing a referring expression in memory through factors like distance from last mention, number of competing referents, and notions of topicality or givenness [17,18]. Pronouns are relatively more accessible compared to full DPs [19], and indeed self-paced reading experiments have revealed faster reading of pronouns as in (1)b compared to definite DPs as in (1)a [20].

(1)
  - George never thinks about how others will feel.
  - He never thinks about how others will feel.

While we target this particular distinction, similar differences can be seen in processing definite compared to indefinite DPs [21].

**FILLER GAP DEPENDENCY**. Another well-known effect in psycholinguistics concerns so-called filler-gap constructions, which feature a grammatical dependency between a fronted element (the filler) and its original syntactic position (the gap). The thematic role of the filler is precisely the one that would have been assigned to the element in the position of the gap. For example, in (2), there is a gap after the main verb *find* that would be filled by a DP. *Which cars* is the filler associated with the gap.

(2) Which cars_i_ did the salesman find _______i_ easiest to sell?

Filler-gap dependencies are claimed to increase processing load because the filler must be held in working memory until the gap is identified, while all the other information encountered must be processed simultaneously [22]. In a landmark study, Wanner & Marastos [23] used a combined comprehension and memory task to test the effects of distance between filler and gap, finding that as distance increases, comprehension decreases. Subsequent studies using self-paced reading tasks have found that in a number of different syntactic constructions, reading times slow precisely where a gap would occur [24,25].

**AGREEMENT ATTRACTION**. The phenomenon of *agreement attraction* describes an instance in which, instead of agreeing in number with its grammatical subject, a verb spuriously agrees with some nearby constituent, as in (3)a–b [26]. A robust finding in the literature is that agreement attraction is much more likely to occur with a nearby attractor that is plural [27]. In other words, sentences like (3)a are much more common than (3)c.

(3)
  - The key to the cabinets are missing.
  - The people who Clark think are in the garden…
  - The keys to the cabinet is missing.

Agreement attraction has been attested in a number of distinct construction types, including with prepositional phrase modifiers [27,28], relative clauses [27,29], auxiliary inversion [30], and *wh*-fronted constructions [31].

## Experiment 1: Replication using AMT

In order to investigate whether pronoun/DP processing effects, filler-gap effects, and agreement attraction can be replicated over the web using AMT, we used stimuli comparable in structure to those in Badecker [31] which make it possible to test all three effects in a single self-paced reading experiment. This experiment was presented to participants using ScriptingRT, as described in more detail below. Self-paced reading tasks typically reveal an increase in reading time at a structure that is difficult to process [32], for example one which is ungrammatical. Following Badecker [31] we used *grammatical* stimuli, with all critical items involving a fronted object *wh*-phrase with an associated gap in post-verbal position, and a DP or pronominal subject. Because attraction effects are expected to occur more often with plural DPs, the number of the subject and *wh*-attractor were also manipulated. These stimuli are schematized in Table 1.

**Table 1:** Test item schema (*t* indicates the gap position).

	*wh*-Attractor	
Subject, Verb	sg.	pl.
sg.	Which X has he/DP verbed *t*_wh_?	Which Xs has he/DP verbed *t*_wh_?
pl.	Which X have they/DP verbed *t*_wh_?	Which Xs have they/DP verbed *t*_wh_?

We make the following general predictions. Reading-times will be slower for DPs compared pronouns, and a slow down in reading-times will occur at or just following a gap. As for attraction effects, these occur in ungrammatical sentences when participants fail to detect erroneous agreement between a verb and an intervening non-subject DP (attractor). However, in grammatical sentences they occur when participants falsely identify as ungrammatical a sentence in which the verb *does not* agree with an attractor. Therefore here agreement attraction effects are predicted to manifest as a slow-down in reading after encountering an attractor DP followed by a verb with agreement that does not match it [28].

### Method

**PARTICIPANTS**. Participants were workers recruited through AMT. In total, 35 external survey HITs were posted. Because of a software problem, one participant received an incomplete test list, therefore data analyzed here come from 34 AMT workers. This sample size is comparable to that used in other studies investigating these same effects [20,21,31]. Two features of our HIT were designed to recruit only participants who were native speakers of English: first we included a *locale qualification* specifying that workers be located in the United States. Second, the informed consent document specified that workers must be native English speakers, have no known language disorders, and be above 18 years of age. Recruiting participants in this way gives up some control (relative to lab-based studies), as confirmation of all these factors is based on self-reporting. However, willfully misrepresenting oneself to complete an AMT HIT is a violation of the worker’s terms of service. Workers were compensated $1.00 for participation in the study, which took approximately 20 minutes. For the sake of comparison with lab-based studies, we were able to recruit and gather data from 35 participants in 7 hours, for a total of $35.00 in payment to participants and $10.50 to AMT ($0.30 per participant).

**ETHICS STATEMENT**. This research was conducted with the approval of the George Mason University human subjects review board. Prior to accepting the HIT, participants were presented with the informed consent document and instructions stating that by clicking “Agree” they indicated their consent to participate in the study. Written consent was waived by the University IRB on the grounds that the research presented no more than minimal risk to participants and the study involved no procedures for which written consent is normally required outside of the research context.

**Fig 1:**
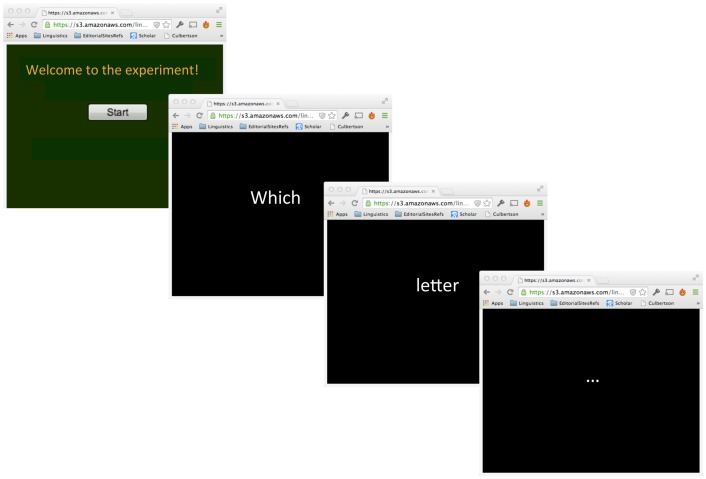
Sample self-paced reading trial. Sample trials of a self-paced reading experiment presented as a Flash movie embedded in HTML page.

**APPARATUS, STIMULI & DESIGN**. The experiment was presented to workers as a Flash movie embedded in an HTML page, as shown in Fig. 1. A link to the wrapper HTML page was posted on AMT as an external survey HIT. Flash is currently a very popular solution to providing dynamic content over the web, and Flash plug-ins are either built into or available for most popular browser applications (Chrome, Firefox, Safari, Internet Explorer). By using Flash to capture response times on the client side, we hope to capture psycholinguistic processing effects with accuracy comparable to that of laboratory software such as Linger [33].

The critical stimuli used in the task consisted of 48 sentence sets arranged in a 2x2x2 design as in Table 1, with *wh*-number (singular, plural), auxiliary/subject number (singular, plural), and subject type (pronoun, DP) manipulated. Stimuli were designed to replicate the structure of the test items used in Badecker [31]. The first two words of each critical item were always the *wh*-phrase, followed by the auxiliary *was* or *were*, then a DP or pronominal subject, then a main (uninflected) verb. Following Badecker [31] an adjective or adverb was added to the sentences with pronominal subjects in order to match the length of the corresponding DP subject stimuli. Example critical stimuli are shown in Table 2. The 48 critical items were combined with 72 filler items, for a total of 120 trials. Fillers were all questions, but did not feature a fronted object *wh*-phrase (e.g., *Was the advertisement for the club colorful*? or *Who paid for the snacks at last month’s meeting*?). This resulted in 60% of the total sentences being filler items (comparable to similar studies, e.g. [29]). The items were distributed among 8 counterbalanced test lists, and each participant was randomly assigned to a list upon accepting the HIT.

**Table 2:** Example stimuli set, Experiment 1.

Stimulus sentence	Subj. Number	*Wh* Number	Subj. Type
(a) Which antique was the maid polishing in the study?	Singular	Singular	DP
(b) Which antique was she polishing in the upstairs study?	Singular	Singular	Pronoun
(c) Which antique were the maids polishing in the study?	Plural	Singular	DP
(d) Which antique were they polishing in the upstairs study?	Plural	Singular	Pronoun
(e) Which antiques was the maid polishing in the study?	Singular	Plural	DP
(f) Which antiques was she polishing in the upstairs study?	Singular	Plural	Pronoun
(g) Which antiques were the maids polishing in the study?	Plural	Plural	DP
(h) Which antiques were they polishing in the upstairs study?	Plural	Plural	Pronoun

**PROCEDURE**. The experiment was a self-paced reading task [32] implemented using ScriptingRT, which compiles into a Flash movie. Workers browsing AMT could see instructions and a link to the consent document. Once a participant accepted the task, a JavaScript function was called to randomly load one of the 8 test lists. The experiment began with on-screen instructions that described the task; participants were told that they would be reading sentences one word at a time, and that pressing the space bar would reveal each subsequent word and hide the previous word. Unlike in a moving-window self-paced reading task, other words in the sentence did not remain on the screen masked. Participants were instructed to read at a natural pace, but slowly enough to comprehend what they read.

Items were randomized using the branching function available through the ScriptingRT library with some additional code written by the first author (available at https://code.google.com/p/enochson-amt/). Reading times were captured in milliseconds for each word. A yes/no comprehension question followed each item (e.g., *Was the antique in the bedroom*?). Participants pressed the “y” key for yes, and the “n” key for no to indicate their response, with feedback given for incorrect responses.

### Results

Data for this and all other experiments reported here are publicly available at http://hdl.handle.net/1920/9116. Sentences for which the participant answered the comprehension question incorrectly were removed from the analyses. It is typical in self-paced reading experiments to discard data from participants who answered more than 20% of the comprehension questions incorrectly or whose reading times are more than 2.5 standard deviations from the mean (e.g., [29]). No participants met either criterion; therefore, analyses were run on all 34 participants. Response times below 100 ms or above 2500 ms were removed; this resulted in loss of between 2% and 2.5% of the data in each of the experiments reported here.

Each sentence can be thought of as comprised of several regions of interest: the *wh* region, the auxiliary region, the subject region, the main verb region, what we will call the V+1 region one word after the verb, and the V+2 region two words after the verb. These regions are illustrated in Table 3 for an example sentence, with regions of most interest highlighted.

**Table 3:** Regions of interest.

*Which cars*	*has*	***the salesman***	***found***	***easiest***	***to***	*sell*?
*Wh*	auxiliary	**subject**	**verb**	**V+1**	**V+2**	

Because the critical regions differed in the number of letters, and in the case of the subject region, in the number of words, all analyses were performed on residual reading times [34]. Log transformed RTs [35] were input into a linear mixed-effects model, using subject number as a random effect, to calculate fitted and residual reading times. Subsequent linear mixed-effects models of residual RTs, using both participant and item as random effects, were used to analyze the effect of each factor of interest and any interactions between them. All models included the maximal random effects structure justified by the data [36]. All statistical modeling and hypothesis testing was performed in R [37], and all mixed-effects models were run using the lme4 package [38].

**Fig 2:**
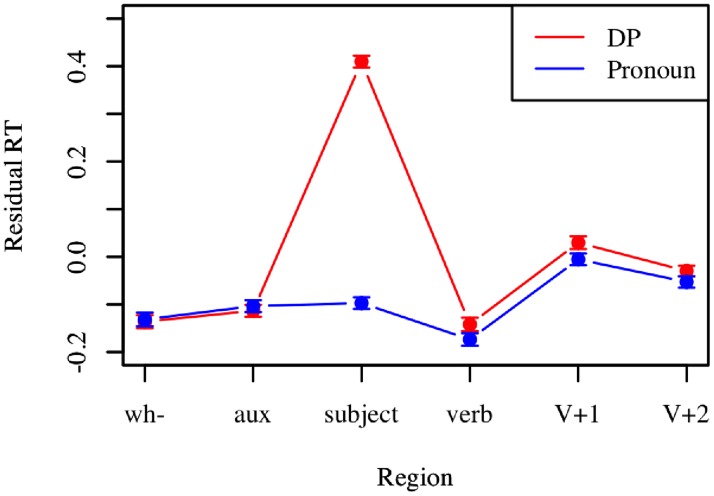
Subject definiteness results. Mean residual reading time is plotted by region for DP and pronoun sentences in Experiment 1. Error bars represent standard error of the mean.

**PRONOUN VS. DP PROCESSING EFFECT**. If subject type (pronoun vs. DP) impacts processing, we expect to see a slow-down in reading time at the subject region for DPs as compared to pronouns. To test whether we can capture this effect using AMT, a linear mixed-effects model was fit using residual reading time as the dependent variable and subject type as a fixed effect. Results indicate that at the subject region, pronouns are read significantly faster than DPs (β = -0.508 ± 0.02, p < 0.0001). This is illustrated in Fig. 2.

**Fig 3:**
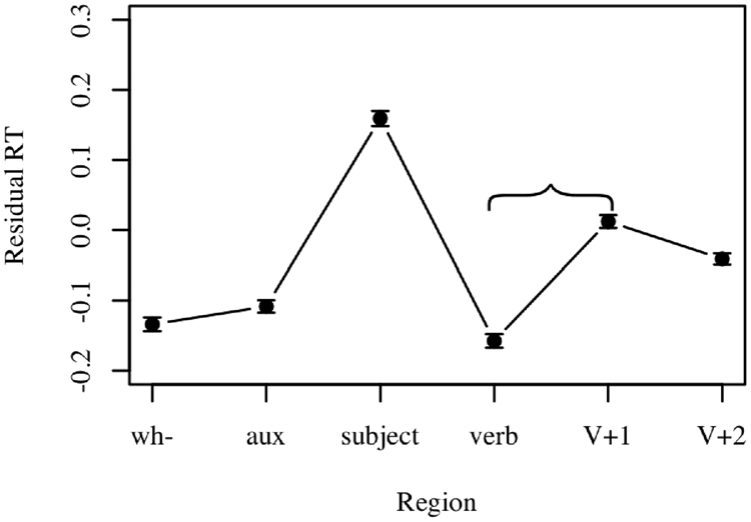
Filler-gap effect results. Mean residual reading time is plotted by region for all conditions in Experiment 1. The effect of interest is the difference between the verb region and the V+1 region. Error bars represent standard error of the mean.

**FILLER-GAP EFFECT**. The filler-gap effect is characterized by increased processing difficulty at the position of a gap. In our case, this predicts a slow-down in reading time following the verb, where the *wh*- phrase gap is filled (e.g., [24,25]). To test whether we have captured this effect, a linear mixed-effects model was fit using residual reading time as the dependent variable, region as the fixed effect, and participant and item as random effects. Tukey-adjusted pairwise comparisons indicate a significant slowdown from the verb region to the V+1 region (β = 0.170 ± 0.02, p < 0.001), indicative of a filler-gap effect. This effect is illustrated in Fig. 3.

**AGREEMENT ATTRACTION EFFECTS**. Recall that for the type of stimuli we are using here—namely grammatical sentences—we expect agreement attraction to present as a slow-down in reading when the subject and verb differ in number from the fronted *wh*-phrase. This would indicate that participants are (erroneously) expecting the verb to agree with the attractor. Here we expect the fronted *wh*-phrase to act as a potential attractor because of its subject-like pre-verbal position in the sentence [31,39]. As mentioned above, agreement attraction is typically found with plural attractors, and thus we also predict a difference in reading-time slow down depending on the number of the *wh*-phrase.

Agreement attraction effects typically spill over from the verb to later regions, (e.g., [28,29]). Therefore, to assess potential agreement attraction effects, linear mixed-effects models were fit for the verb region, the V+1 region, and the V+2 region, using residual reading time as the dependent variable, and a binary factor coding number mismatch between subject and *wh* number as the fixed effect. Number mismatch was not significant in any of the regions. Since agreement attraction is *more* likely when the subject is singular and the attractor is plural, as discussed above, it could be that agreement attraction is found only in the latter case. A significant interaction between *wh* number and mismatch was indeed found in the verb region, however it did not persist into any of the spillover regions. To assess whether pronoun subjects might block agreement attraction effects (by providing a particularly strong cue to subjecthood), a model was also fit using subject type and number mismatch as fixed effects. This interaction was not significant in any region. Estimates and p-values for all models are shown in Table 4.

**Table 4:** Estimates and (p-values) for agreement attraction models. Bold font indicates a significant result.

	Effect	Verb region	V+1 region	V+2 region
Model 1.	Mismatch	-0.002 (0.91)	-0.004 (0.87)	-0.013 (0.47)
Model 2.	*wh*-number * mismatch	**0.075 (0.04)**	0.039 (0.25)	0.020 (0.58)
Model 3.	subject type * mismatch	0.020 (0.60)	0.005 (0.89)	0.005 (0.87)

The significant slowdown in reading time found when the subject was singular and the attractor plural suggests that we may have uncovered evidence of agreement attraction. However, the fact that the effect was found only in the verb region and not in any later spillover regions contrasts with previous findings of agreement attraction in other construction types (e.g., [29]). We address this issue in detail below.

### Interim summary

The experiment reported here used a single set of stimuli to test whether three important psycholinguistic effects could be replicated with response time data gathered over the web via AMT. The first two effects—the pronoun vs. DP processing difference and the filler-gap effect—were successfully replicated, using a similar number of participants and items as a more traditional lab-based study. The third effect—agreement attraction—was not convincingly replicated. This could potentially be related to the particular construction we used, or to the fact that we used only grammatical stimuli. While other researchers (e.g., [28]) have found attraction effects in grammatical sentences in production and self-paced reading studies, Pearlmutter et al. [28] suggest that the magnitude of attraction effects in grammatical sentences is generally smaller than in ungrammatical sentences. Badecker [31] did report attraction effects using grammatical *wh*-questions, however our methodology was not the same; Badecker [31] used a production task rather than a comprehension task. Since no other studies demonstrate agreement attraction using grammatical *wh*-questions in a self-paced reading task, it may be that attraction in this context is not as robust or reliable as some other contexts. Additionally, although Experiment 1 used a comparable number of participants to other studies of filler-gap dependency and subject definiteness (e.g., [20,21]), the number is low compared to other agreement attraction studies (e.g., [28,29]). In Experiments 2 and 3, we seek to replicate agreement attraction using structures that have consistently demonstrated robust effects, specifically prepositional phrase modifiers and relative clause modifiers, using the same number of participants as the corresponding laboratory studies.

## Experiment 2: Further Investigations of Agreement Attraction

Experiment 2 attempts to replicate the agreement attraction effects reported in Pearlmutter et al. [28]. Here we focus on agreement attraction in sentences with prepositional phrase modifiers, e.g., *The slogan on the poster(s) was/were designed to get attention*. In such sentences, the DP contained in the prepositional phrase intervenes between the subject and the agreeing verb, potentially triggering agreement attraction [27–30,40].

### Method

**PARTICIPANTS**. In order to match as closely as possible the task reported in Pearlmutter et al. [28], we recruited an identical number of participants, namely 82. These participants were recruited and compensated via AMT in the same manner as in Experiment 1 over the course of one week.

**ETHICS STATEMENT**. This research was conducted with the approval of the George Mason University human subjects review board. Prior to accepting the HIT, participants were presented with the informed consent document and instructions stating that clicking “Agree” indicates voluntary participation. Written consent was waived by the University’s IRB on the grounds that the research presented no more than minimal risk to participants and the study involved no procedures for which written consent is normally required outside of the research context.

**APPARATUS, STIMULI & DESIGN**. As in Experiment 1, this experiment was presented to workers as a ScriptingRT Flash movie embedded in an HTML page. Stimuli for Experiment 2 were taken from Pearlmutter et al. Experiment 1 [28]. All critical stimuli had singular subjects (since attraction is typically most likely with plural attractors), and we manipulated attractor number and sentence grammaticality. A sample stimuli set is shown in Table 5, where the most likely context for agreement attraction should be sentences of type (d). As in Pearlmutter et al. [28], there were 16 critical test items and 94 filler items for a total of 110 trials. Stimuli were distributed among 4 counterbalanced test lists to which participants were randomly assigned.

**Table 5:** Example stimuli set, Experiment 2.

Stimulus	Attractor Number	Grammaticality
(a) The slogan on the poster was designed to get attention.	singular	grammatical
(b) The slogan on the posters was designed to get attention.	plural	grammatical
(c) The slogan on the poster were designed to get attention.	singular	ungrammatical
(d) The slogan on the posters were designed to get attention.	plural	ungrammatical

**PROCEDURE**. HITs were posted to AMT in the same manner as Experiment 1, and the self-paced reading task procedure was identical. This differs slightly from Pearlmutter et al., [28], who use a moving window design.

### Results and Discussion

Sentences for which the participant answered the comprehension question incorrectly were removed from the analyses. As in Experiment 1, no participants answered more than 20% of the comprehension questions incorrectly, and no participants had reading times more than 2.5 standard deviations from the mean. Therefore, analyses were run on all 82 participants. Note that Pearlmutter et al., [28] ultimately excluded 2 from the analyses due to low comprehension question performance, therefore our replication includes data from two additional participants. Data were processed in the same manner as in Experiment 1.

**Fig 4:**
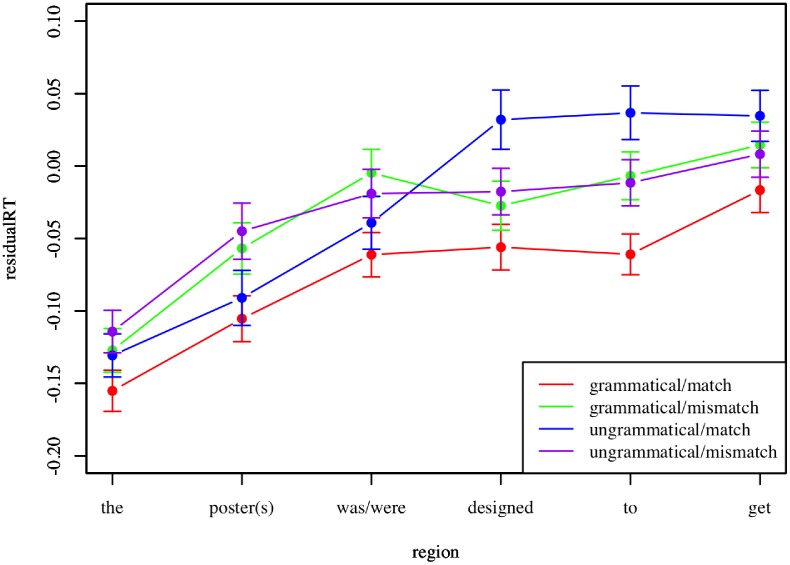
Agreement attraction with PP modifiers. Mean residual reading time is plotted by region for each condition in Experiment 2. Error bars represent standard error of the mean.

Recall that our stimuli included both grammatical and ungrammatical sentences. In general, grammatical sentences should be read faster than ungrammatical sentences. However, if the intervening DP in the prepositional phrase serves as an agreement attractor, then we expect a decrease in reading time associated with ungrammaticality in sentences that include a number mismatch between the subject and the verb. Put another way, since all subjects are singular, ungrammatical sentences with a plural attractor should be read faster than ungrammatical sentences with a singular attractor. To investigate this, a linear mixed-effects model was fit using residual reading time as the dependent variable, grammaticality and number mismatch as fixed effects, and participant and item number as random effects. Pearlmutter et al. [28] report a spill-over agreement attraction effect two words after the verb. Given the example sentence *The slogan on the poster(s) was/were designed to get attention*, we would then expect agreement attraction to present as a slow down at the word “to”. A simple effect of ungrammaticality should occur at the verb, with effects potentially spilling over onto the next two regions. Our data reveal a significant interaction between ungrammaticality and number mismatch at the region corresponding with the word “to” (β = -0.09 ± 0.03, p = 0.005), such that when an item is ungrammatical and the subject number and attractor number do not match, the item is read faster. This indicates a successful replication of the study in Pearlmutter et al. [28] and is illustrated in Fig. 4.

Experiment 2 demonstrates that agreement attraction effects can be captured using AMT, indicating that our failure to capture such effects in Experiment 1 is likely a function of the stimuli rather than the method. Experiment 3 attempts to extend this result, replicating attraction effects reported in Wagers et al. [29] using relative clause modifiers.

## Experiment 3

In Experiment 3 we attempt to replicate agreement attraction effects in relative clause modifiers reported in Wagers et al. [29]. For example, in a sentence like *The runner(s) who the driver see(s) during the commute every morning always wave(s) to say hi*, the main clause subject *runner(s)* functions as a potential attractor for the agreeing relative clause verb *see(s)*. Because the original task as described in Wagers et al. [29] is substantially longer than Experiments 1 and 2 (a total of 192 items compared to 120 and 110 respectively), here we reduce the number of items and increase the number of subjects.

### Method

**PARTICIPANTS**. Wagers et al. [29] used data from 30 participants; we doubled this to 60 participants, recruited and compensated as in Experiments 1 and 2. Participants were recruited over the course of 4 days.

**ETHICS STATEMENT**. This research was conducted with the approval of the George Mason University human subjects review board. Prior to accepting the HIT, participants were presented with the informed consent document and instructions stating that clicking “Agree” indicates voluntary participation. Written consent was waived by the University’s IRB on the grounds that the research presented no more than minimal risk to participants and the study involved no procedures for which written consent is normally required outside of the research context.

**APPARATUS, STIMULI & DESIGN**. As in Experiments 1 and 2, this experiment was presented to workers as a ScriptingRT Flash movie embedded in an HTML page. Stimuli for Experiment 3 come from Wagers et al. Experiment 2 [29]. As mentioned above, in order to keep Experiment 3 consistent with Experiments 1 and 2 in terms of time and compensation, we used a subset of the items; in particular we used the first half of the critical items from the Wagers et al. stimuli, and half the number of filler items. Thus we had 24 critical items and 72 fillers, resulting in 96 total items. All items used singular subjects, and attractor number and grammaticality were manipulated. A sample stimuli set is shown in Table 6, where agreement attraction is expected to be most likely in sentences of type (d). The stimuli were distributed among 4 counterbalanced test lists to which participants were randomly assigned.

**Table 6:** Example stimuli set, Experiment 3.

Stimulus	Attractor Number	Grammaticality
(a) The runner who the driver sees during the commute. . .	singular	grammatical
(b) The runners who the driver sees during the commute. . .	plural	grammatical
(c) The runner who the driver see during the commute. . .	singular	ungrammatical
(d) The runners who the driver see during the commute. . .	plural	ungrammatical

**PROCEDURE**. HITs were posted to AMT in the same manner as Experiments 1 and 2, and the self-paced reading task procedure was identical. This differs slightly from Wagers et al. [29], who use a moving window design.

### Results and Discussion

Sentences for which the participant answered the comprehension question incorrectly were removed from the analyses. One participant was excluded for having mean reading times more than 2.5 standard deviations from the mean. No participants answered more than 20% of comprehension questions incorrectly. Therefore, analyses were run on 59 participants. Data were processed in the same manner as in Experiments 1 and 2.

**Fig 5:**
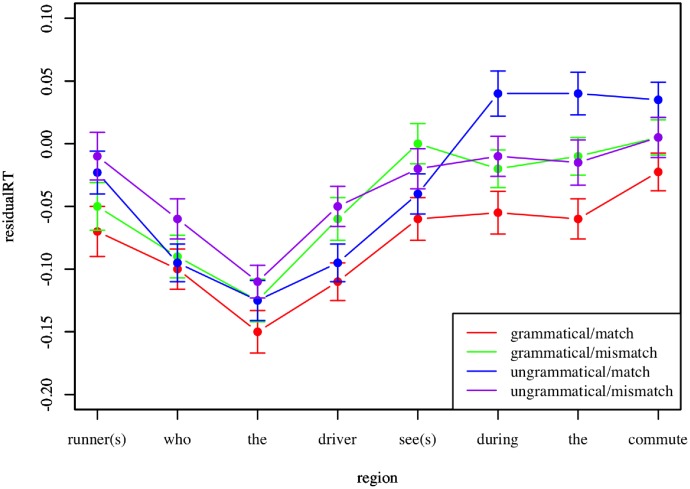
Agreement attraction with RC modifiers. Mean residual reading time is plotted by region for each condition in Experiment 3. Error bars represent standard error of the mean.

As in Experiment 2, agreement attraction should lead to a decrease in reading time associated with ungrammaticality in sentences that include a number mismatch between the subject and the verb. To assess this, a linear mixed-effects model was fitted using residual reading time as the dependent variable, grammaticality and number mismatch as fixed effects, and participant and item number as random effects. The region of interest in these stimuli is two words after the relative clause ends, so in the example sentence *The runner(s) who the driver see(s) during the commute*…, agreement attraction would likely present at the word “the” in “the commute”. Ungrammaticality occurs at the verb “see(s)”, and again effects should spill over onto the next two regions. Our data reveal a significant interaction between ungrammaticality and number mismatch at the region corresponding with the word “the” (β = -0.02 ± 0.008, p = 0.001), indicating agreement attraction effects. This is illustrated in Fig. 5.

## General Discussion

### Summary

In this paper, we have demonstrated that AMT can be successfully used to conduct psycholinguistic research in which precise response time measurements are necessary. The effects we replicated here involve self-paced reading tasks designed to reveal differences in the processing of pronouns vs. DPs, filler-gap effects, and agreement attraction. While we were not able to convincingly find agreement attraction effects in grammatical *wh*-fronted questions, we were able to replicate agreement attraction in two more established contexts—with prepositional phrase and relative clause modifiers. Importantly, we used similar numbers of trials and participants as traditional lab studies; our three experiments used between 35–82 participants each, and between 96–120 trials each, well within the typical range for self-paced reading and other psycholinguistic tasks. In terms of the resources used, the cost to our lab of running these experiments was $1.30 per participant ($1 to the participant, $0.30 to AMT), and each experiment was completed within a week or less.

### Comparing AMT data with lab data

As mentioned above, the major concern associated with using web-based methods to gather behavioral data is a potential increase in variability. In addition to the higher variability that might result from a more diverse population of participants, in the case of response time data there are also differences in keyboard sampling and/or monitor refresh rates across devices. Because even the fastest keyboard sampling rates can never capture a time *faster* than the participant’s response time, an increase in keyboard sampling rate variability will result in an overall increase in mean response times. It is worth noting that issues of variable keyboard sampling rate and monitor refresh rate are not unique to web-based research methods; across laboratories using different computers and keyboards or even within a laboratory with multiple computers, the same issues arise. However, there is reason to suspect that the magnitude of hardware differences is not large enough to impact qualitative results. Further, in the case of AMT, the variety of different computer monitors and keyboards should essentially wash out any effect of differences in refresh rate and sampling rate in the aggregate.

A number of other studies have compared data gathered in the lab to data gathered over AMT in qualitative [2,3,5,9] and quantitative [41] terms. These studies suggest that data captured over the web can successfully replicate data gathered in the lab. However, as our goal is to replicate psycholinguistic effects using response time data gathered over AMT, we provide here a direct comparison with laboratory results. We provide this for Experiment 1, since Experiments 2 and 3 are close replications of previously published lab-based studies. Participants were 22 undergraduate students from George Mason University who completed the study in exchange for extra credit in an undergraduate linguistics course. The experiment was presented using the Linger software [33]. The materials and procedure were otherwise identical to Experiment 1 as reported above.

Results of our laboratory replication of Experiment 1 reveal the same significant findings—subject definiteness and filler-gap effects—as the AMT study, and similarly fail to capture agreement attraction in grammatical *wh*-fronted constructions. Mixed-effect regression models for these data are summarized in Table 7.

**Table 7:** Estimates and (p-values) for laboratory replication of Experiment 1. Bold font indicates a significant result.

Effect	Subject region	Verb region	V+1 region	V+2 region
Subject type	**-0.728 (0.000)**	**---**	---	---
Filler-gap effect	---	---	**0.131 (0.018)**	---
Mismatch	---	-0.020 (0.47)	-0.006 (0.78)	-0.024 (0.24)
*wh*-number * mismatch	---	-0.099 (0.07)	0.006 (0.89)	-0.062 (0.22)
subject type * mismatch	---	0.045 (0.41)	0.036 (0.40)	0.017 (0.71)

Interestingly, mean response times and standard error for the lab data were *larger* than those captured using AMT, even when comparing lab data to the first 22 AMT participants; reading times in the lab are an average of 180ms slower and standard error is 5ms greater. It is perhaps not surprising that AMT participants complete experiments faster than laboratory participants; AMT participants are unmonitored and are essentially paid by the hour, incentivizing them to work as quickly as possible on each task. By comparison, laboratory participants are typically monitored by an experimenter, and in our case were compensated with course credit.

In sum then, the experiments reported here using AMT were largely successful in replicating several robust psycholinguistic processing effects using a self-paced reading task. Moreover, in the case of Experiment 1—which failed to replicate agreement attraction effects with fronted *wh-*phrases—findings from AMT were qualitatively identical to those gathered in the lab. In this subsection we discuss some aspects of the experimental design, specific to AMT, which we believe may increase the likelihood of successful replication of lab-based results.

### Recommendations for future use of AMT in psycholinguistics

**MASTER’S QUALIFICATION**. Sprouse [2] notes in his large-scale replication of acceptability judgment results that the participant rejection rate was substantially higher over AMT than in the lab. In all the experiments reported here we required workers to have *Master’s qualification*. This qualification is reserved for participants who have completed a large number of HITs from a variety of requesters with a high level of accuracy. This is not a qualification that workers can apply for; rather, it is awarded based on statistical monitoring of acceptance rates by Amazon, and workers must maintain a high level of performance in order to keep the qualification. Notable, out of the 326 participants we ran, only 1 needed to be excluded for performance-related reasons (e.g., too many incorrect comprehension questions or reading times greater than 2.5 standard deviations from the mean). We can report anecdotally that this is lower than subsequent self-paced reading studies that we have run on AMT in which we did not require Master’s qualification. Moreover, it is lower than other published lab-based studies using self-paced reading tasks (2/30 in [29] and 2/82 in [28]). Using workers with Master’s qualification costs more than using regular workers; for Master’s workers, the requester (researcher) pays Amazon an additional $0.20 per dollar. This means that our experiments cost $1.30 per participant, compared to a total cost of $1.10 per participant for workers without Master’s qualification. If there is concern about data quality and minimization of data loss, however, we believe it is worth the additional cost.

**BATCH-POSTING HITS**. When a batch of HITs is posted by a requester on AMT, it appears as a single entry at the top of the workers’ list of available HITs. As time passes, newer HITs get posted, and older HITs appear lower on the list, becoming less likely to be selected by workers. Our experience suggests that posting HITs in large batches significantly increases the time it takes to get results. For example, in one case posting a batch of 30 altogether took about a week to complete, while posting 6 batches of 5 took approximately six hours. For all the experiments reported here we posted HITs in batches of 5. We recommend this as a way to further increase the speed with which data can be gathered over AMT.

**CODING ISSUES**. We have argued here that using AMT to conduct psycholinguistics experiments minimizes the time and cost relative to traditional lab-based methods. Nevertheless, programming experiments in which response time data are recorded does require some specialized knowledge. In particular, while AMT provides templates for several basic experiment types (e.g., Likert scale judgment tasks and translation tasks), self-paced reading, visual priming, or serial response time tasks require use of either Flash/ActionScript or JavaScript. Here we have used ScriptingRT, a free and open source software program for collecting this type of data. An alternative is to use JavaScript (in fact our lab is currently using JavaScript for a number of experiments, and example code can be found at https://code.google.com/p/enochson-amt/). The crucial feature of any software used is that it capture response times on the client side to avoid issues related to server variability. As researchers begin work with these types of tasks over the web, it is likely that modifications of this or other code will be necessary, and we believe that it is of crucial importance that researchers continue to make code publicly available.

## Conclusion

Our goal in this paper was to contribute to the body of existing evidence validating the use of Amazon Mechanical Turk, and web-based methods more generally, to collect precise response time data for psycholinguistic research. Using a self-paced reading task displayed as a Flash movie embedded in a HTML webpage, we successfully replicated three important psycholinguistic effects: a difference in processing pronouns compared to DPs (definiteness effects), the processing cost of filler-gap constructions, and agreement attraction (in prepositional phrase and relative clause modifiers). Importantly, our replications used sample sizes and numbers of items similar to the original experiments, with results nevertheless qualitatively matching data collected in traditional lab studies. While some degree of control over participants and the specific devices they use is relinquished when using AMT, there was little if any evidence to suggest that this impacted our ability to uncover the effects of interest. Importantly, the time and cost of our AMT replication studies was a fraction of what is typically required to conduct research in psycholinguistics. Web-based methods for data collection like AMT therefore represent an important resource-effective tool for moving the field forward, and we hope the current work encourages researchers to make use of it.

## References

[1] Gibson E, Piantadosi S, Fedorenko K (2011) Using Mechanical Turk to obtain and analyze English acceptability judgments. Lang Linguist Compass 5: 509–524.

[2] Sprouse J (2011) A validation of Amazon Mechanical Turk for the collection of acceptability judgments in linguistic theory. Behav Res Methods 43: 155–167. 10.3758/s13428-010-0039-7 21287108 PMC3048456

[3] Schnoebelen T, Kuperman V (2010) Using Amazon mechanical turk for linguistic research. Psihologija 43: 441–464.

[4] Fine AB, Jaeger TF, Farmer TA, Qian T (2013) Rapid Expectation Adaptation during Syntactic Comprehension. PLoS ONE 8: e77661 10.1371/journal.pone.0077661 24204909 PMC3813674

[5] Munro R, Bethard S, Kuperman V, Lai VT, Melnick R, et al (2010) Crowdsourcing and language studies: the new generation of linguistic data. Proceedings of the NAACL HLT 2010 Workshop on Creating Speech and Language Data with Amazon’s Mechanical Turk. Association for Computational Linguistics. pp. 122–130. Available: http://dl.acm.org/citation.cfm?id=1866715. Accessed 13 August 2014.

[6] Culbertson J, Adger D (2014) Language learners privilege structured meaning over surface frequency. Proc Natl Acad Sci 111: 5842–5847. 10.1073/pnas.1320525111 24706789 PMC4000832

[7] Becker M, Nevins A, Levine J (2012) Asymmetries in generalizing alternations to and from initial syllables. Language 88: 231–268.

[8] Henrich J, Heine SJ, Norenzayan A (2010) The weirdest people in the world? Behav Brain Sci 33: 61–83. 10.1017/S0140525X0999152X 20550733

[9] Crump MJC, McDonnell JV, Gureckis TM (2013) Evaluating Amazon’s Mechanical Turk as a Tool for Experimental Behavioral Research. PLoS ONE 8: e57410 10.1371/journal.pone.0057410 23516406 PMC3596391

[10] Keller F, Gunasekharan S, Mayo N, Corley M (2009) Timing accuracy of web experiments: A case study using the WebExp software package. Behav Res Methods 41: 1–12. 10.3758/BRM.41.1.12 19182118

[11] Fine AB, Florian Jaeger T (2013) Evidence for Implicit Learning in Syntactic Comprehension. Cogn Sci 37: 578–591. 10.1111/cogs.12022 23363004

[12] Demberg V (2013) Integration Costs on Auxiliaries?–a self-paced reading study using WebExp. Available: http://csjarchive.cogsci.rpi.edu/Proceedings/2013/papers/0396/paper0396.pdf. Accessed 14 October 2014.

[13] Drummond A (2013) Ibex Farm. Available: http://spellout.net/ibexfarm/. Accessed 2014 October 14.

[14] Schmidt S (2009) Shall we really do it again? The powerful concept of replication is neglected in the social sciences. Rev Gen Psychol 13: 90.

[15] Klein RA, Ratliff KA, Vianello M, Adams RB Jr, Bahník Š, et al (2014) Investigating Variation in Replicability. Soc Psychol 45: 142–152.

[16] Schubert TW, Murteira C, Collins EC, Lopes D (2013) ScriptingRT: A Software Library for Collecting Response Latencies in Online Studies of Cognition. PLoS ONE 8: e67769 10.1371/journal.pone.0067769 23805326 PMC3689727

[17] Ariel M (1990) Accessing noun-phrase antecedents. Croom Helm Lond.

[18] Gundel JK, Hedberg N, Zacharski R (1993) Cognitive status and the form of referring expressions in discourse. language: 274–307.

[19] Gordon PC, Grosz BJ, Gilliom LA (1993) Pronouns, names, and the centering of attention in discourse. Cogn Sci 17: 311–347.

[20] Warren T, Gibson E (2002) The influence of referential processing on sentence complexity. Cognition 85: 79–112. 12086714 10.1016/s0010-0277(02)00087-2

[21] Hofmeister P, Sag IA (2010) Cognitive Constraints and Island Effects. Language 86: 366–415. 22661792 10.1353/lan.0.0223 PMC3364522

[22] Hawkins JA (1999) Processing complexity and filler-gap dependencies across grammars. Language: 244–285.

[23] Wanner E, Marastos M (1978) An ATN approach to comprehension. Linguist Theory Psychol Real. Available: http://ci.nii.ac.jp/naid/10008829763/. Accessed 2014 May 8.

[24] Crain S, Fodor JD (1985) How can grammars help parsers? Natural language parsing: Psychological, computational, and theoretical perspectives. pp. 94–128.

[25] Stowe LA (1986) Parsing WH-constructions: Evidence for on-line gap location. Lang Cogn Process 1: 227–245.

[26] Kimball J, Aissen J (1971) I think, you think, he think. Linguist Inq 2: 241–246.

[27] Bock K, Miller CA (1991) Broken agreement. Cognit Psychol 23: 45–93. 10.1016/0010-0285(91)90003-7 2001615

[28] Pearlmutter NJ, Garnsey SM, Bock K (1999) Agreement processes in sentence comprehension. J Mem Lang 41: 427–456.

[29] Wagers MW, Lau EF, Phillips C (2009) Agreement attraction in comprehension: Representations and processes. J Mem Lang 61: 206–237.

[30] Vigliocco G, Nicol J (1998) Separating hierarchical relations and word order in language production: Is proximity concord syntactic or linear? Cognition 68: B13–B29. 9775519 10.1016/s0010-0277(98)00041-9

[31] Badecker W (in prep) Agreement in Wh-Questions: Subject-oriented Retrieval Mechanisms in a Working-Memory Retrieval Model of Sentence Production.

[32] King J, Just MA (1991) Individual differences in syntactic processing: The role of working memory. J Mem Lang 30: 580–602. 10.1016/0749-596X(91)90027-H

[33] Rohde D (2003) Linger: a flexible platform for language processing experiments, version 2.94.

[34] Ferreira F, Clifton C (1986) The independence of syntactic processing. J Mem Lang 25: 348–368.

[35] Baayen RH, Milin P (2010) Analyzing reaction times. Int J Psychol Res 3: 12–28.

[36] Barr DJ, Levy R, Scheepers C, Tily HJ (2013) Random effects structure for confirmatory hypothesis testing: Keep it maximal. J Mem Lang 68: 255–278. 10.1016/j.jml.2012.11.001 PMC3881361 24403724

[37] R Core Team (2014) R: A language and environment for statistical computing. Vienna, Austria: R Foundation for Statistical Computing Available: http://www.R-project.org/. Accessed 1 March 2014.

[38] Bates D, Maechler M, Bolker B (2012) lme4: Linear mixed-effects models using S4 classes. Available: http://www.citeulike.org/group/17044/article/12173300. Accessed 10 September 2014.

[39] MacWhinney B, Bates E, Kliegl R (1984) Cue validity and sentence interpretation in English, German, and Italian. J Verbal Learn Verbal Behav 23: 127–150.

[40] Solomon ES, Pearlmutter NJ (2004) Semantic integration and syntactic planning in language production. Cognit Psychol 49: 1–46. 15193971 10.1016/j.cogpsych.2003.10.001

[41] Germine L, Nakayama K, Duchaine BC, Chabris CF, Chatterjee G, et al (2012) Is the Web as good as the lab? Comparable performance from Web and lab in cognitive/perceptual experiments. Psychon Bull Rev 19: 847–857. 22829343 10.3758/s13423-012-0296-9

